# New Challenges to Study Heterogeneity in Cancer Redox Metabolism

**DOI:** 10.3389/fcell.2017.00065

**Published:** 2017-07-11

**Authors:** Rui Benfeitas, Mathias Uhlen, Jens Nielsen, Adil Mardinoglu

**Affiliations:** ^1^Science for Life Laboratory, KTH Royal Institute of Technology Stockholm, Sweden; ^2^Department of Biology and Biological Engineering, Chalmers University of Technology Gothenburg, Sweden

**Keywords:** cancer heterogeneity, redox biology, reactive oxygen species, systems biology, personalized medicine

## Abstract

Reactive oxygen species (ROS) are important pathophysiological molecules involved in vital cellular processes. They are extremely harmful at high concentrations because they promote the generation of radicals and the oxidation of lipids, proteins, and nucleic acids, which can result in apoptosis. An imbalance of ROS and a disturbance of redox homeostasis are now recognized as a hallmark of complex diseases. Considering that ROS levels are significantly increased in cancer cells due to mitochondrial dysfunction, ROS metabolism has been targeted for the development of efficient treatment strategies, and antioxidants are used as potential chemotherapeutic drugs. However, initial ROS-focused clinical trials in which antioxidants were supplemented to patients provided inconsistent results, i.e., improved treatment or increased malignancy. These different outcomes may result from the highly heterogeneous redox responses of tumors in different patients. Hence, population-based treatment strategies are unsuitable and patient-tailored therapeutic approaches are required for the effective treatment of patients. Moreover, due to the crosstalk between ROS, reducing equivalents [e.g., NAD(P)H] and central metabolism, which is heterogeneous in cancer, finding the best therapeutic target requires the consideration of system-wide approaches that are capable of capturing the complex alterations observed in all of the associated pathways. Systems biology and engineering approaches may be employed to overcome these challenges, together with tools developed in personalized medicine. However, ROS- and redox-based therapies have yet to be addressed by these methodologies in the context of disease treatment. Here, we review the role of ROS and their coupled redox partners in tumorigenesis. Specifically, we highlight some of the challenges in understanding the role of hydrogen peroxide (H_2_O_2_), one of the most important ROS in pathophysiology in the progression of cancer. We also discuss its interplay with antioxidant defenses, such as the coupled peroxiredoxin/thioredoxin and glutathione/glutathione peroxidase systems, and its reducing equivalent metabolism. Finally, we highlight the need for system-level and patient-tailored approaches to clarify the roles of these systems and identify therapeutic targets through the use of the tools developed in personalized medicine.

## Introduction

**Figure 1:**
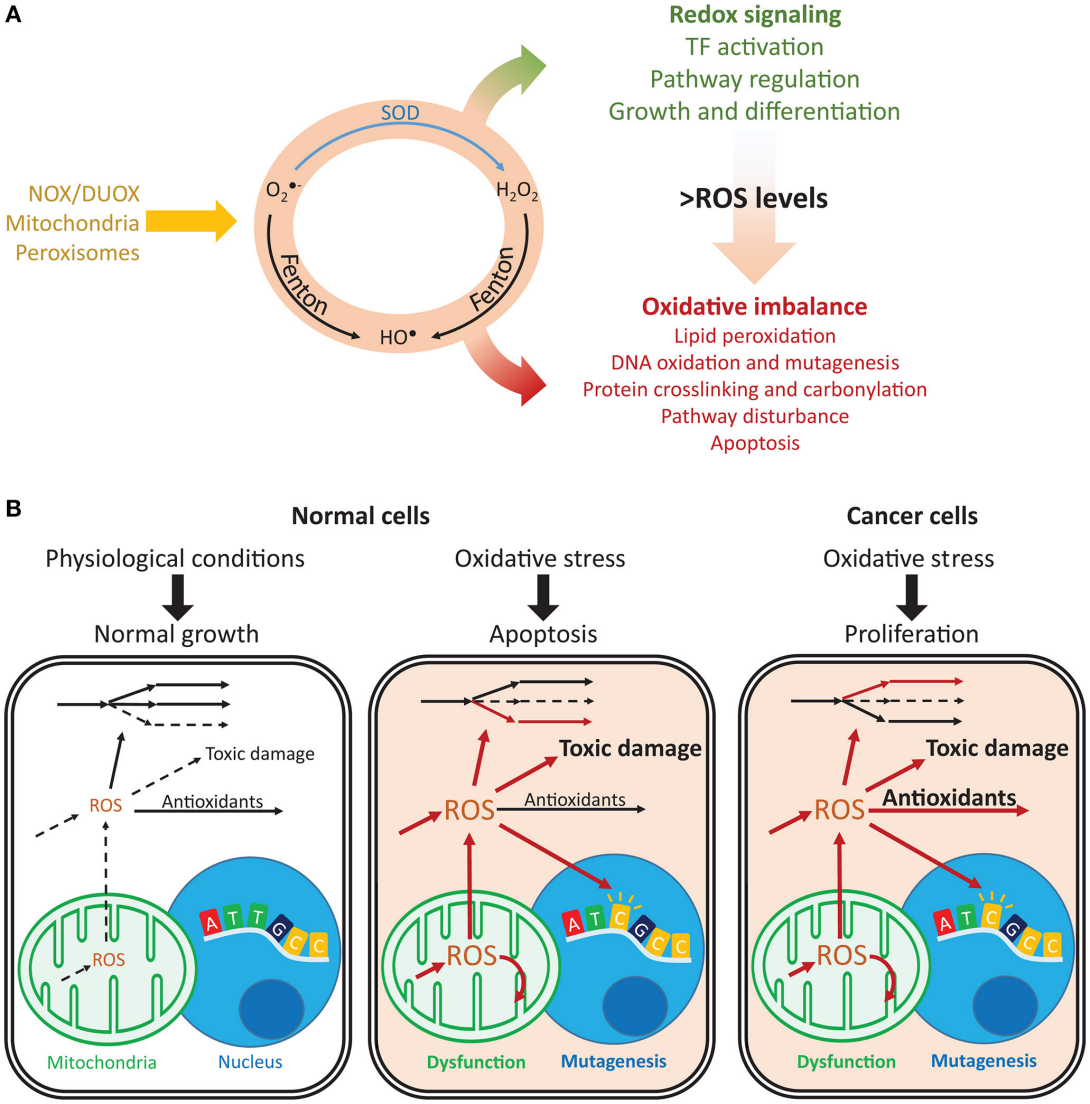
Imbalances in ROS and redox cycles lead to contrasting outcomes in normal and cancer cells. **(A)** ROS are intracellularly produced by NADPH oxidases and dual oxidases (NOX/DUOX) through mitochondrial oxidative phosphorylation and in peroxisomes. Their interconversion (orange circle) occurs through enzyme catalyzed and non-catalyzed reactions. For instance, metal-catalyzed Fenton reactions produce HO^•^ from ${\text{O}}_{2}^{•-}$ and H_2_O_2_. Under low ROS levels, these oxidants control important signaling reactions, activating transcription factors, regulating pathways and controlling cell growth and differentiation. Under high ROS levels, oxidation of lipids, nucleic acids, and proteins is toxic and may disturb pathways and lead to cell death. **(B)** Normal and cancer cells present important differences in their responses to oxidative stress. Under normal conditions, ROS production is low and antioxidant defenses are sufficient to prevent toxic damage. Under oxidative stress, the promoted production of ROS overcomes the cell's capacity for detoxification and results in increased toxic damage and pathway disruption, which may lead to mitochondrial dysfunction, mutagenesis and ultimately apoptosis. In turn, cancer cells are under constant oxidative stress, which through upregulation of antioxidant defenses, prevents apoptosis while maintaining ROS toxicity. Arrows indicate fluxes, increasing from dashed to continuous, in red. ${\text{O}}_{2}^{•-}$, superoxide; H_2_O_2_, hydrogen peroxide; HO^•^, hydroxyl radical; SOD, superoxide dismutase.

Redox metabolism is closely intertwined with cell physiology, and reactive oxygen species (ROS) are central players in health and disease. For instance, these oxygen-derived species are involved in cancer (Reuter et al., 2010), neurodegenerative diseases (Sultana et al., 2006), aging (Höhn et al., 2013), and diabetes (Evans et al., 2002). They are produced intracellularly by several processes and dedicated enzymes, such as NADPH oxidases (Nauseef, 2008) and in multiple cellular compartments (Messner and Imlay, 2002; Chen et al., 2008; Murphy, 2009; Brown and Borutaite, 2012). Due to their high membrane permeability (Chance et al., 1979), extracellularly produced ROS (Hampton et al., 1998; Babior et al., 2002) may quickly enter cells, or they may diffuse across compartments (Bienert et al., 2007; Marchissio et al., 2012). In many diseases, imbalances in ROS metabolism lead to oxidative stress. As result, cells face toxic outcomes of protein, lipid, and nucleic acid oxidation (Garrison, 1987; Cooke et al., 2003; Smith and Murphy, 2008; Figure 1A). For instance, DNA oxidation by ROS promotes mutagenesis, cancer initiation, and progression (Shibutani et al., 1991; Cooke et al., 2003; Sabharwal and Schumacker, 2014), and at high concentrations, ROS may cause cell apoptosis (Gao et al., 2013).

In turn, low ROS concentrations have important physiological roles (D'Autréaux and Toledano, 2007). They regulate cell-cycle progression (Havens et al., 2006), proliferation (Choe et al., 2012), growth (Arnold et al., 2001), and important signaling processes (Finkel, 2011; Rigoulet et al., 2011). For instance, hydrogen peroxide (H_2_O_2_) regulates the activity of kinases (Gotoh and Cooper, 1998; Paulsen et al., 2012), which control proliferation, differentiation, and apoptosis. NFE2L2 (also known as NRF2) responds to oxidative stress and regulates GSH biosynthesis and reduction, the expression of several proteins involved in antioxidant defense (glutathione peroxidases, transferases, peroxiredoxins, thioredoxins, and thioredoxin reductases), and NADPH production (Gorrini et al., 2013). Many of these redox-regulated processes are not directly controlled by ROS but rather by their redox partners. For instance, redox signaling transduction is often mediated by peroxiredoxins, thioredoxins, and other thiol-reacting proteins (Saitoh et al., 1998; Giannoni et al., 2005; Morinaka et al., 2011). These proteins ensure the high specificity required for efficient signaling transduction (Nagy and Winterbourn, 2010; Winterbourn, 2013; Marinho et al., 2014; Netto and Antunes, 2016). Together with other cellular antioxidants, such as catalases and dismutases, these redox systems prevent toxic ROS accumulation while permitting special-temporal selectivity and the maintenance of important redox signaling functions.

In cancer, mitochondrial dysfunction and metabolic changes promote constant oxidative stress (Szatrowski and Nathan, 1991; Hileman et al., 2004); however, this does not result in apoptosis. Cancer cells promote the expression of antioxidant defenses or reducing equivalents that enable their activity (Janssen et al., 1999; Miranda et al., 2000; Hileman et al., 2004), thus avoiding ROS-induced apoptosis and enabling proliferation, despite high mutagenesis (Toyokuni et al., 1995; Kondo et al., 1999) and metastasis (Ishikawa et al., 2008; Figure 1B). ROS closely interact with iron (Galaris et al., 2008) and central (Robbins et al., 2012; Hart et al., 2015; Miar et al., 2015) metabolism, and they are controlled by several transcription factors and tumor suppressors (Gao et al., 2007; Frohlich et al., 2008; Gupta et al., 2012; Gorrini et al., 2013; Hornsveld and Dansen, 2016). Additionally, antioxidant enzymes may display high or low expression in cancer cells (Ray et al., 2000; Oltra et al., 2001; Skrzydlewska et al., 2005; Glorieux et al., 2015), suppress tumorigenesis or promote metastasization (Zhao et al., 2001; Liu et al., 2012; Robbins et al., 2012; Miar et al., 2015), and display synergistic responses (Harris et al., 2015). As result, systematic approaches may capture these complex responses and provide insights into the mechanisms underlying the diverse phenotypic responses in cancer.

Systems biology presents promising approaches for capturing and studying complex cellular responses (Mardinoglu et al., 2013b; Ghaffari et al., 2015b). The application of such frameworks to clinical challenges is referred to as systems or network medicine (Mardinoglu and Nielsen, 2012, 2016). The complexity of biological pathways in cells and tissues may be captured through reconstruction of biological networks, including genome-scale metabolic models (GEMs), transcriptional regulatory networks, protein–protein interaction networks, and signaling networks, in an integrated approach that aims to understand entire cell processes at the systems level (Mardinoglu and Nielsen, 2015; Zhang et al., 2016). These networks may also be integrated with each other for a holistic understanding of the relationships between cellular networks, function, and disease (Bjornson et al., 2016; Lee et al., 2016; Mardinoglu and Uhlén, 2016). Generation of omics data for major human tissues enabled the generation of comprehensive biological networks (Kampf et al., 2014a; Lindskog et al., 2015; Uhlén et al., 2015, 2016; Thul et al., 2017), which have been successfully employed in revealing the underlying mechanisms involved in the occurrence of obesity (Mardinoglu et al., 2013a, 2014b, 2015a), type 2 diabetes (Väremo et al., 2015), non-alcoholic fatty liver disease (Kampf et al., 2014b; Mardinoglu et al., 2014a, in press; Hyötyläinen et al., 2016), and cancer (Agren et al., 2012, 2014; Weinstein et al., 2013; Zack et al., 2013; Leiserson et al., 2014; Yizhak et al., 2014b; Aran et al., 2015; Bjornson et al., 2015; Peng et al., 2015; Elsemman et al., 2016). Personalized models have also been used in the identification of potential therapeutic targets and biomarkers (Faratian et al., 2009; Agren et al., 2014; Bjornson et al., 2015; Mardinoglu et al., 2017; Nielsen, 2017). To date, small scale redox networks have also been analyzed (Zhang et al., 2010; Zhou et al., 2011; Zhan et al., 2012). However, despite extensive evidence highlighting the importance of ROS, antioxidants and other redox players in cancer, systems approaches have yet to systematically examine the role of redox metabolism in this disease and uncover potential personalized treatment strategies.

Here, we highlight some recent findings about important biological processes that are crucial in tumorigenesis: ROS, their redox partners, and reducing equivalents. We start by overviewing some of the main biochemical properties of ROS and their effectors. We then discuss the role of antioxidants and their reactions in tumorigenesis, focusing on thiols and reducing equivalents due to their importance in ROS and redox homeostasis. Finally, given the high heterogeneity of redox responses and the intricate crosstalk between redox and central metabolism, we highlight how system-level and patient-tailored approaches may help to identify potential cancer targets and provide mechanistic insights into redox cancer responses. These discussions do not aim to be exhaustive descriptions of all biological processes and regulators of redox homeostasis, and the interested reader may find excellent reviews on these topics elsewhere (e.g., Gao et al., 2007; Frohlich et al., 2008; Gupta et al., 2012; Gorrini et al., 2013; Hornsveld and Dansen, 2016).

## Biochemistry of ROS and redox systems

Molecular oxygen freely diffuses across cell membranes and promotes the formation of intracellular ROS through electron abstraction. ROS may be classified as radicals and non-radicals. Radicals have unpaired electrons and include superoxide (${\text{O}}_{2}^{•-}$) and the hydroxyl (HO^•^) radicals. Non-radical ROS do not have unpaired electrons, and they include H_2_O_2_. Here, we focus on these three ROS due to their patho-physiological importance. ROS metabolism yields many other less reactive, abundant, or stable ROS and is highly intertwined with other important reactive species (such as Reactive Nitrogen Species, Weidinger and Kozlov, 2015).

ROS are formed in several intracellular compartments. Most notably, they are produced in peroxisomes through fatty acid oxidation (Fransen et al., 2012), in mitochondria during oxidative phosphorylation and in the cellular and intracellular membranes by NADPH oxidases (EC 1.6.3.1; Nauseef, 2008; Kowaltowski et al., 2009; Murphy, 2009; Brown and Borutaite, 2012; Fransen et al., 2012). Some crosstalk exists between these systems. For instance, mitochondrial-produced ROS promote ${\text{O}}_{2}^{•-}$ generation by NADPH oxidases, which may have important functions during phagocytosis (Dikalov, 2011). It is currently unclear which of the compartments above contributes the most to intracellular ROS production, although mitochondria are often cited as the main cellular ROS source (Brown and Borutaite, 2012).

**Figure 2:**
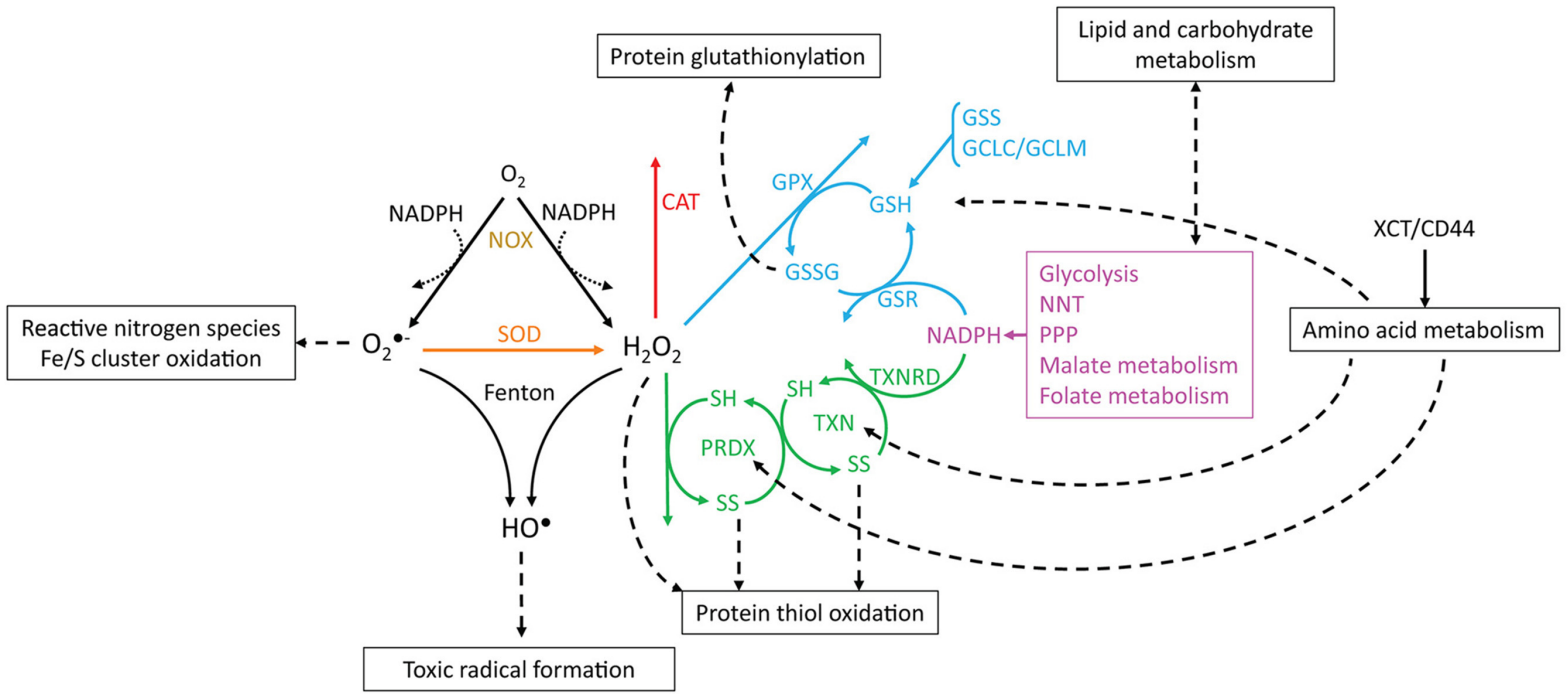
Reactions involving ROS, antioxidant systems and energy metabolism. ${\text{O}}_{2}^{•-}$ and H_2_O_2_ are produced from oxygen through reactions that may oxidize NADPH (dotted arrows, e.g., catalysis by NADPH oxidases). ${\text{O}}_{2}^{•-}$ is dismutated to H_2_O_2_ by SOD, and ${\text{O}}_{2}^{•-}$ and H_2_O_2_ may be converted to HO^•^ by Fenton reactions. CAT, PRDX and GPX scavenge H_2_O_2_. The catalytic cycles of PRDX and TXN are represented, where SH and SS, respectively, indicate reduced and oxidized (disulfide) thiols. Boxes and dashed arrows indicate the external processes with which the metabolites are associated. For example, XCT/CD44 mediates cysteine import, which may then be incorporated into proteins, such as TXN and PRDX, or may be metabolized to yield GSH. Colors indicate proteins or processes from the same pathway.

The reactions involving ROS and their cellular targets lead to the interconversion of various types of ROS (Figure 2). For instance, mitochondrial- and cytoplasmic-produced ${\text{O}}_{2}^{•-}$ is dismutated to H_2_O_2_ by superoxide dismutases (SOD, EC 1.15.1.1). ${\text{O}}_{2}^{•-}$ is fairly unreactive to most electron-rich centers due to its anionic charge, but it reacts with nitric oxide to form peroxynitrite (Huie and Padmaja, 1993) and oxidizes iron-sulfur clusters, thereby producing H_2_O_2_ and HO^•^ (Rouault and Klausner, 1996). These clusters are found in multiple intracellular compartments (Tong et al., 2000), and their oxidation by ${\text{O}}_{2}^{•-}$ (or H_2_O_2_) leads to iron release and the inactivation of metabolically important enzymes, such as those involved in amino acid biosynthesis (Wallace et al., 2004) or carbohydrate metabolism (Gardner et al., 1995). It is currently unclear whether most ${\text{O}}_{2}^{•-}$ is used toward nitric oxide metabolism, if it reacts with metal clusters, or if it is dismutated to H_2_O_2_. Both dismutases and nitric oxide react with ${\text{O}}_{2}^{•-}$ with near diffusion-limited rate constants (*k* > 10^9^ M^−1^ s^−1^, Bannister et al., 1973; Huie and Padmaja, 1993), and ${\text{O}}_{2}^{•-}$ is very reactive with some iron-sulfur cluster-bearing enzymes (*k* ≈ 10^6^–10^7^ M^−1^ s^−1^, Flint et al., 1993), but it is unreactive with amino acid residues (Bielski and Shiue, 1979). The fate of ${\text{O}}_{2}^{•-}$ depends on the local availability of the other reactants or enzymes and likely varies between cells and under different conditions, although it is generally assumed that most ${\text{O}}_{2}^{•-}$ is dismutated to H_2_O_2_ (Forman et al., 2010).

In turn, H_2_O_2_ reacts slowly with most biological compounds, such as free glutathione (*k* < 10 M^−1^ s^−1^, Winterbourn and Metodiewa, 1999) and phosphatases (*k* ≈ 10–200 M^−1^ s^−1^, LaButti et al., 2007; Marinho et al., 2014). However, it may display extremely high reactivities with selected protein thiols due to their neighboring chemical environment (*k* ≈ 10^5^–10^8^ M^−1^s^−1^, Peskin et al., 2007; Trujillo et al., 2007; Manta et al., 2009). It is decomposed into water and molecular oxygen as a result of dismutation by catalases (EC 1.11.1.6), or it is reduced to water by peroxidases and peroxiredoxins (EC 1.11.1.15). Often, several of these mechanisms are present in the same cells. Protection against H_2_O_2_ is accomplished through glutathione peroxidase, catalase, and peroxiredoxin 2 in human erythrocytes (Johnson et al., 2005; Low et al., 2007; Benfeitas et al., 2014). These defenses are unlikely to be redundant in their functions: while catalase is an efficient H_2_O_2_ scavenger, even under high oxidative loads, peroxiredoxin 2 has limited reduction under such conditions (Low et al., 2007), resulting in a lower contribution for H_2_O_2_ consumption. However, peroxiredoxin 2 and its coupled cycles display desirable redox signaling properties (Benfeitas et al., 2014), which are further discussed below.

${\text{O}}_{2}^{•-}$ and H_2_O_2_ are also involved in the production of HO^•^ through iron-catalyzed Fenton reactions involving heme peroxidases or iron/sulfur clusters (Fenton, 1894; Chen and Schopfer, 1999; Koppenol, 2001). Iron accumulation and HO^•^ production have been extensively associated with carcinogenesis, and iron chelators have been employed as therapeutic drugs in cancer (reviewed by Torti and Torti, 2013; Bystrom and Rivella, 2015). Due to its electrophilic nature, HO^•^ preferably oxidizes electron-rich sites, reacting with nucleic acids, lipids, and proteins with diffusion-limited rate constants (Von Sonntag, 1987; Buxton et al., 1988; Stadtman and Levine, 2003; Sharma and Rokita, 2013). This promotes DNA strand breaks, lipid peroxidation and protein carbonylation, crosslinking, and cleavage. The products of these reactions are toxic, and they often promote radical propagation and damage to nearby molecules through subsequent chain reactions. Importantly, due to HO^•^'s very high and unselective reactions with biological compounds, no cellular antioxidants can feasibly scavenge this oxidant before it reacts with cellular contents. Instead, protection against the toxic outcomes of HO^•^ comes from preventing its formation by shielding iron from ROS or by scavenging H_2_O_2_ and ${\text{O}}_{2}^{•-}$ before they yield HO^•^. The role of H_2_O_2_ in HO^•^ formation and consequential radical formation is also thought to be one of the main reasons behind H_2_O_2_'s toxicity (Winterbourn, 1995).

**Figure 3:**
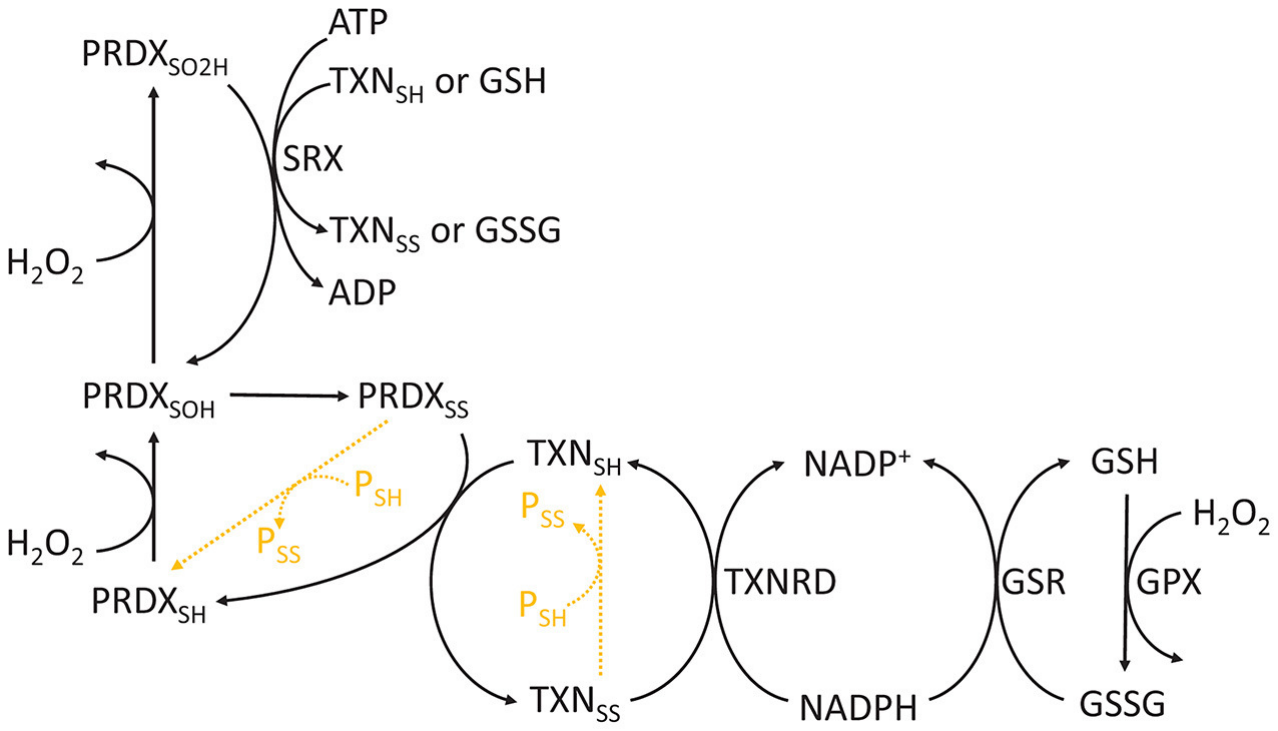
Chemical cycle of PRDXs, TXNs, and GSH. The redox state of PRDXs and TXN is indicated as follows: SH, reduced cysteine thiol; SOH, sulfenic acid; SO_2_H, sulfinic acid; SS, disulfide. Orange dashed reactions highlight disulfide exchange between peroxiredoxins and thioredoxins and other proteins.

As result of their different reactivities with biological compounds, the above ROS present varying stabilities and cellular roles. For instance, the fast and indiscriminate reactions of HO^•^ result in very small diffusion distances (≈80 Å, Roots and Okada, 1975) that are approximately the size of a small peptide. For this reason, HO^•^ is likely to oxidize molecules near its formation site (estimated half-life of 10^−9^ s, Pryor, 1986), and it is unfit to behave as a signaling molecule. ${\text{O}}_{2}^{•-}$ is also regarded as a poor signaling effector because it does not permeate cell membranes and is quickly dismutated to H_2_O_2_, or it reacts with iron/sulfur clusters and nitric oxide. This results in low intracellular stability, hindering its diffusion across large distances. Therefore, while ${\text{O}}_{2}^{•-}$ has some regulatory properties, these properties are possibly due to its role in nitric oxide and H_2_O_2_ metabolism (Brune, 2005; Thomas et al., 2006; D'Autréaux and Toledano, 2007; Kaewpila et al., 2008; Labunskyy and Gladyshev, 2013). In turn, H_2_O_2_ has emerged as the ROS that displays the best signaling properties. Its high stability and selective reactions with cellular compounds permit diffusion over distances of several micrometers (Winterbourn, 2008) and enable cell membrane crossing, which is also facilitated through specific channels (Bienert et al., 2007). H_2_O_2_ reacts with cellular thiols, including those contained in low molecular weight compounds, such as glutathione, and protein thiols, such as peroxiredoxins and thioredoxins (Box 1 and Figure 3). These reactions convert an oxidizing equivalent into a redox signal, which may be transduced from protein to protein via thiol disulfide exchange or between glutathione and proteins, forming mixed disulfides. Together with intracellular thiols, H_2_O_2_ regulates the redox state and activity of several target proteins and has pivotal importance in both physiological and pathological conditions (D'Autréaux and Toledano, 2007). Due to this intricate association, any discussion about the involvement of ROS in tumorigenesis also needs to consider the role of thiols and other antioxidant defenses. Although these systems have been widely studied in cancer, the role of ROS, antioxidant defenses, and redox signaling transducing partners in cancer is only now emerging beyond antioxidant activities. Further ROS-centered studies aimed at clarifying these properties and their involvement in cancer are still required.

BOX 1 **Thiols as important redox signaling sensors and effectors.**

Cysteine's thiol side chains (R-SH) are often very reactive with H_2_O_2_. They undergo a series of reversible or irreversible redox transitions, which are represented here through the catalytic cycles of a typical 2-Cys peroxiredoxin/thioredoxin (Figure 3). In this cycle, the reduced form is subsequently oxidized by H_2_O_2_ to sulfenic (R-SOH) and sulfinic (R-SO_2_H) acids. The sulfinic form may be irreversibly oxidized to sulfonic (R-SO_3_H) forms *in vitro*, but it is currently unclear whether this process occurs *in vivo*. Sulfinic acids may be reduced to sulfenic acids by specific proteins (e.g., sulfiredoxins, SRX) at the expense of ATP and the oxidation of TXN and GSH (Chang et al., 2004). Sulfenic acids may also conjugate to form intra- or inter-molecular disulfide bonds (R-SS-R'). Disulfides are then reduced at the expense of reducing equivalents, such as those found in NADPH (e.g., oxidized GSH or thioredoxin reduction by reductases), or by disulfide exchange with other proteins. Therefore, cysteine oxidation by H_2_O_2_ may be transduced to partner proteins or small molecular weight compounds.

Due to their chemical and kinetic properties, the systems above have potentially different signaling properties. Although they exhibit slow reactivities with H_2_O_2_ when isolated (*k* ≈ 2.9 M^−1^ s^−1^ for free Cys, Winterbourn and Metodiewa, 1999), some cysteine thiols display extremely high reactivities (*k* ≈ 10^5^–10^8^ M^−1^ s^−1^ for peroxiredoxins, Trujillo et al., 2007; Manta et al., 2009). GSH is very abundant, but it is relatively unreactive with H_2_O_2_ *per se*, so the kinetics of glutathione peroxidases should be considered when assessing glutathione's intracellular role in H_2_O_2_ detoxification and signaling. These differences in reactivity also manifest within the same pathway. Reduced and sulfenic forms quickly react with H_2_O_2_, unlike sulfinic and sulfonic acids, which are relatively unreactive with H_2_O_2_. The process of thiol oxidation to disulfide exchange may transduce oxidative equivalents to target proteins, as observed in the proteins above (Jarvis et al., 2012; Naticchia et al., 2013; Sobotta et al., 2015). For instance, thiol-disulfide exchange between peroxiredoxins and phosphatases/kinases is a mechanism for explaining H_2_O_2_-induced signaling regulation despite the low reactivity of H_2_O_2_ with phosphatases/kinases (Ray et al., 2012; Marinho et al., 2014; Sobotta et al., 2015; Latimer and Veal, 2016). Importantly, peroxiredoxin-mediated disulfide exchange controls the activity of several proteins involved in cancer (Park et al., 2007; Jarvis et al., 2012; Sobotta et al., 2015), which reinforces the role of H_2_O_2_ and redox metabolism in this disease. Similar to disulfide exchange, the oxidation of glutathione may lead to S-glutathionylation of proteins, which also regulates their activities (e.g., peroxiredoxin 2, Peskin et al., 2016). These and other properties possibly explain the involvement of PRDXs and TXNs in redox signaling, and they point toward these proteins as good redox sensors and signaling transducers (Benfeitas et al., 2014; Latimer and Veal, 2016; Netto and Antunes, 2016; Tomalin et al., 2016).

It should be noted that many of these redox systems depend on reducing equivalents to maintain their activity. Reducing equivalents are any molecules that act as electron donors in reactions, typically in reference to NADH and NADPH. These species are used by several enzymes, such as reductases, which couple their oxidation to the reduction of thioredoxin or glutathione. This provides another possible layer of selectivity in redox homeostasis: should NADPH utilization be prioritized toward one system over another, the physiological role of the former would also be prioritized over the latter. Interestingly, the link between energy metabolism and redox metabolism goes beyond NAD(P)H-enabling reductase activity. For instance, glyceraldehyde-3-phosphate dehydrogenase, an NADH-producing enzyme essential to glycolysis, is inactivated by glutathionylation and H_2_O_2_-induced disulfide formation (Little and O'brien, 1969; Mohr et al., 1999). ROS and redox-coupled processes thus not only consume reducing equivalents but also regulate energy metabolism.

## ROS as oncogenic drivers and targets of therapeutic strategies

**Figure 4:**
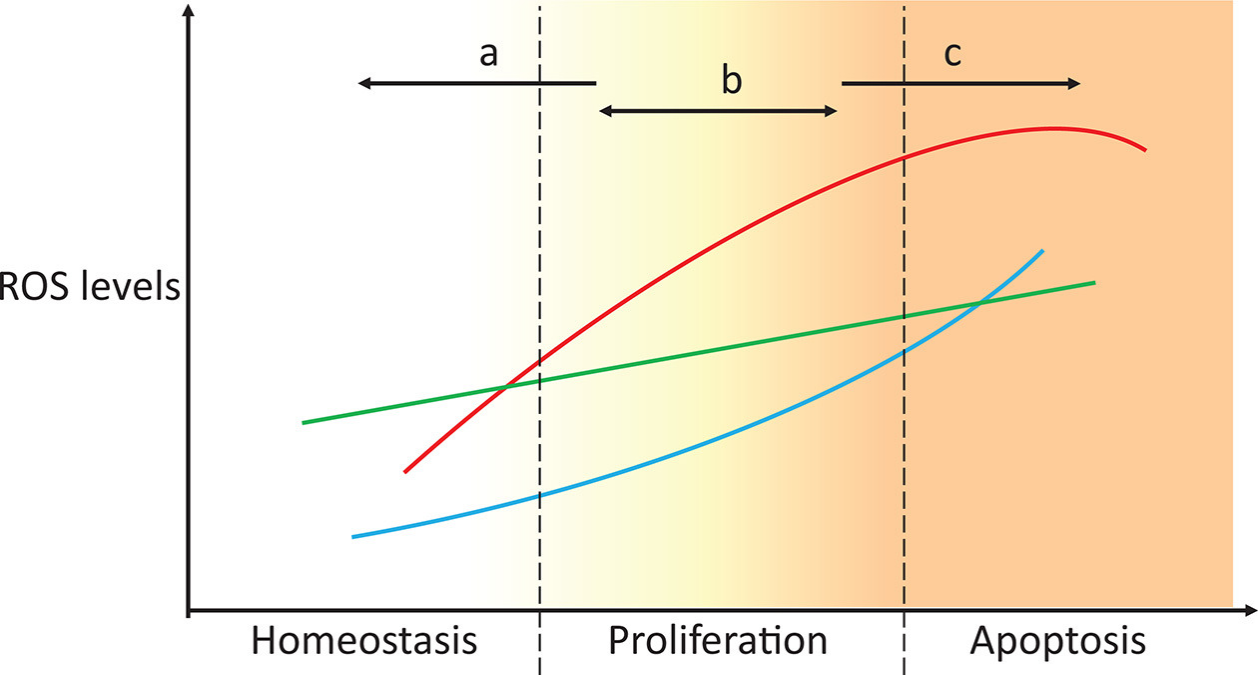
Targeting ROS homeostasis as a strategy for changing cell fate. In proliferative conditions, such as cancer, targeting antioxidant systems could move the redox state of the cell to either promote normal redox homeostasis or apoptosis (strategies a and c, respectively). Unsuccessful tackling of antioxidant metabolism results in the cells maintaining a proliferative state, which potentially enhances malignancy. The three lines represent the high heterogeneity between individuals, tissues, and cancer types.

The role of ROS in cell physiology is highly dependent on their levels. Under physiological levels, ROS regulate a number of signaling processes by reacting with proteins, genes, and transcription factors. ROS control adaptation to hypoxia, regulation of differentiation, immunity, and longevity (Sena and Chandel, 2012). However, the accumulation of ROS beyond physiological levels promotes cell proliferation, angiogenesis, and even apoptosis (D'Autréaux and Toledano, 2007; Cairns et al., 2011; Figure 4), and ROS also control cell-cycle progression (Menon and Goswami, 2007). Oncogene-induced senescence promotes AMP-activated protein kinase activation, mitochondrial dysfunction and ROS production, which trigger senescence, thereby forming a positive feedback loop. Cancer cells display high ROS production (Szatrowski and Nathan, 1991; Ray et al., 2000), which is often also associated with antioxidant imbalances (Skrzydlewska et al., 2005). This results in damage to nuclear (Shibutani et al., 1991) and mitochondrial DNA (Ishikawa et al., 2008; Weinberg et al., 2010). Mutations in nucleic acids may be particularly toxic for the cell if they occur in tumor suppressors or oncogenes. DNA mutations (Higinbotham et al., 1992; Du et al., 1994), in turn, promote ROS generation, thereby resulting in a vicious cycle of ROS production and mutagenesis concomitant with high proliferation. Mitochondrial-generated ROS are also essential for tumor aggressiveness and metastasis (Ishikawa et al., 2008; Weinberg et al., 2010; Goh et al., 2011), and increased cytoplasmic and mitochondrial ROS levels are observed in metastatic nodules and circulating tumors when compared to subcutaneous tumors (Piskounova et al., 2015).

ROS also stabilize the factors that drive tumor initiation and progression (Gao et al., 2007), and promote protein oxidation and the formation of toxic protein carbonyls (Stadtman and Levine, 2003). Protein carbonylation is an irreversible process present in cancer cells (Thanan et al., 2012). Carbonyls may propagate to other proteins or lipids, which may result in the formation of toxic byproducts through chain reactions. ROS-induced lipid peroxidation products, such as 4-hydroxy-2-nonenals, also have multiple physiological roles under low levels, but they become toxic upon accumulation. These species also accumulate in cancer cells (Skrzydlewska et al., 2005; Ayala et al., 2014; Zhong and Yin, 2015).

Due to the toxic effects of ROS accumulation in cancer cells and the fact that some ROS responses may be exclusive to cancer cells but not to their healthy counterparts (Hileman et al., 2004), antioxidants were envisioned as potentially important drug targets in cancer treatment. Initial ROS-focused clinical trials aimed to prevent ROS accumulation (Figure 4, strategy a). In the Linxian study (Blot et al., 1993) stomach cancer patients supplemented with selenium, vitamin E and β-carotene exhibited lower mortality. However, antioxidant-supplemented diets often failed to yield significant changes in cancer development, and in some cases, these diets even promoted tumorigenesis and metastasis (Omenn et al., 1996; Goodman et al., 2011; Klein et al., 2011; Sayin et al., 2014). This is because cancer cells often cope with increased ROS production by increasing the levels of antioxidant defenses or reducing equivalents that maintain their activity (Weinberg et al., 2010; DeNicola et al., 2011). This effect is also observed in metastases, where metastatic melanoma nodules are exposed to additional oxidative stress that is not observed in established subcutaneous tumors, and the nodules cope with this stress by promoting the expression of multiple NADPH-producing pathways (Piskounova et al., 2015). High antioxidant activities enable fast ROS-driven proliferation and metastasization, but the increased oxidative stress is insufficient to lead to apoptosis (Figure 4, strategy b). Higher antioxidant expression is also associated with the radioresistance observed in certain cancer stem cell populations (Diehn et al., 2009). In turn, recent antioxidant-targeted therapeutic strategies have shifted their focus in the opposite direction, exploiting ROS toxicity as a means leading to cancer cell apoptosis (Figure 4, strategy c). These drugs often mimic ROS-generating enzymes (e.g., NADPH oxidases and superoxide dismutases), inhibit antioxidant enzymes (e.g., catalase), deplete thiol pools, such as GSH, or shift redox buffer ratios (e.g., GSSG/2 GSH), thereby promoting a more oxidizing intracellular state and cell apoptosis. These strategies have been discussed in great detail in recent reviews (Ushio-Fukai and Nakamura, 2008; Gupta et al., 2012; Gorrini et al., 2013; Tong et al., 2015).

**Figure 5:**
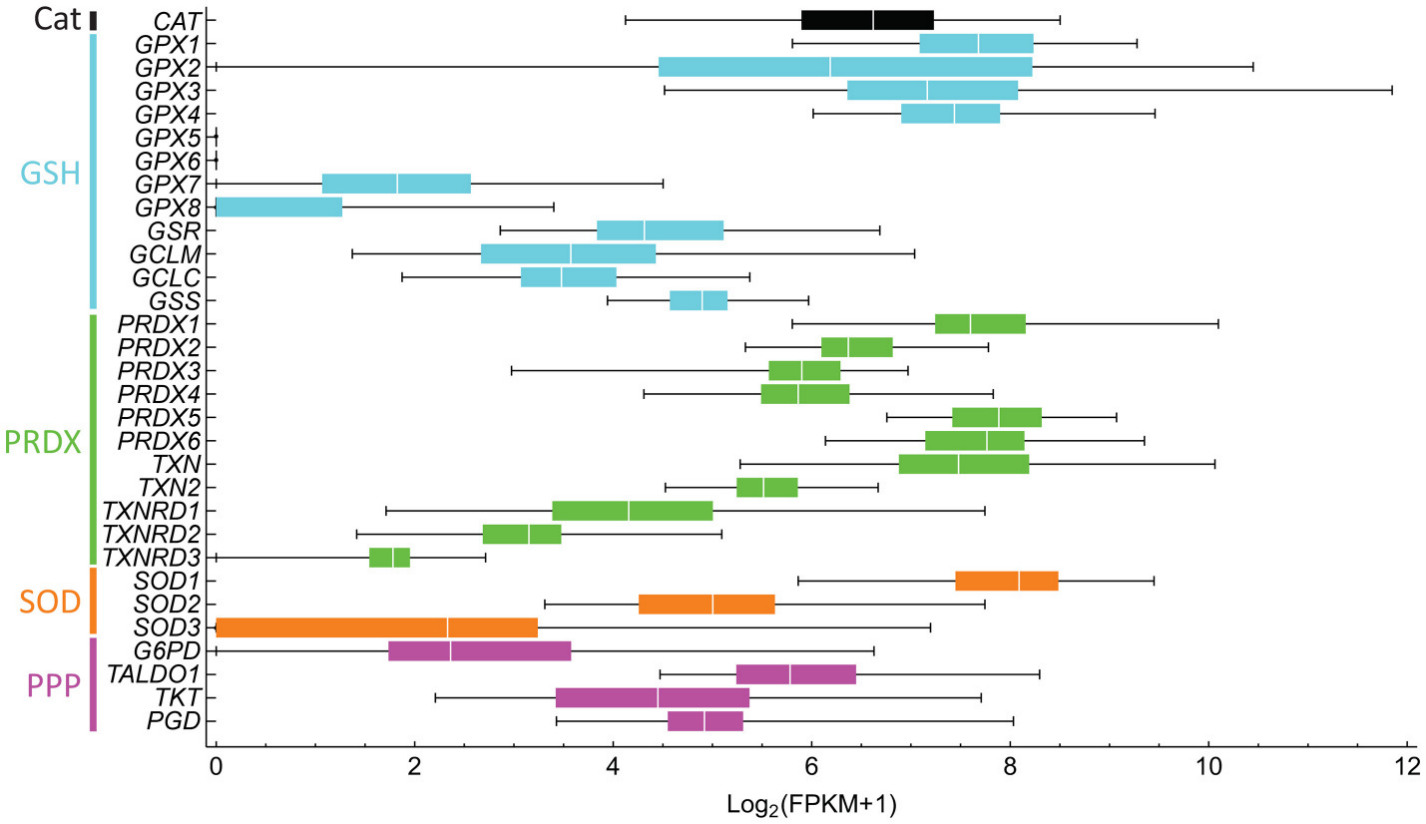
Antioxidant gene expression greatly varies between liver hepatocellular carcinoma in different subjects. The gene expression of 50 subjects was downloaded from NCI's Genomic Data Commons, and fragments per kilobase transcript per million (FPKM) were computed. FPKM-values lower than one were considered to be non-expressed and were assigned a value of 0. The Log_2_(FPKM + 1) were then computed. Bars are colored according to processes of the same pathway, as indicated on the left. Genes and respective proteins: CAT, catalase; GPX1-8, glutathione peroxidase; GSR, glutathione reductase; GCLM and GCLC, glutamate-cysteine ligase modifier and catalytic subunits, respectively; GSS, glutathione synthetase; PRDX1-6, peroxiredoxin; TXN and TXN2, thioredoxin; TXNRD1-3, thioredoxin reductase; SOD, superoxide dismutase; G6PD, glucose-6-phosphate dehydrogenase; TALDO1, transaldolase 1, TKT, transketolase, and PGD, 6-phosphogluconate dehydrogenase.

The targets of many of these drugs are not completely understood, nor is it known whether the drugs are targeting the best redox effectors. Are the targeted compounds selective in treating cancer, but not healthy cells? Are they the best target in that pathway? Which combined drug treatments could improve treatment? These questions stem from an insufficient understanding of redox metabolism in tumorigenesis, which is greatly caused by the high variability in metabolic and redox responses. For instance, different antioxidant defenses that target the same ROS are up- and down-regulated in cancer cells (Skrzydlewska et al., 2005), complicating the interpretation of their role and that of their target ROS in cancer progression. This high variability is even observed between individuals with the same cancer type (Figure 5). Furthermore, genes involved in the same processes are differentially expressed. For instance, a high dispersion in gene expression levels is observed within peroxiredoxins (*PRDX1-6*), thioredoxins (*TXN* and *TXN2*), and thioredoxin reductases (*TXNRD1-3*), which are highly conserved protein systems involved in H_2_O_2_ scavenging and redox signaling transduction. As result of this variability, redox-focused therapeutic strategies (Figure 4) must consider patient-specific data to determine the best approach.

## Role of antioxidant defenses in cancer

Imbalances in antioxidant defenses are one of the hallmarks of cancer (Oberley and Oberley, 1997; Huang et al., 2000; Chung-man Ho et al., 2001; Hu et al., 2005; Murawaki et al., 2008). For instance, increased expression of *SOD* has been observed in multiple cancers (Ray et al., 2000; Skrzydlewska et al., 2005; Holley et al., 2012; Miar et al., 2015) and metastatic tissues (Miar et al., 2015). Its overexpression promotes carcinogenesis (Lu et al., 1997) and aggressiveness (Hempel et al., 2011). In turn, *SOD* deficiency is associated with a higher cancer incidence and DNA damage in mice (Van Remmen et al., 2003; Elchuri et al., 2005), and it has also been observed in some cancers (Oltra et al., 2001). *SOD* levels fluctuate throughout the cell cycle and regulate growth factor cancer signaling (Nelson et al., 2003; Juarez et al., 2008), cell cycle progression, and the energetic changes of cells upon cancer transformation (Hempel et al., 2011; Sarsour et al., 2014). Catalases, often regarded as the main cellular defenses against H_2_O_2_ in human cells, may be up- or down-regulated in cancer cells (Ray et al., 2000; Oltra et al., 2001; Skrzydlewska et al., 2005; Glorieux et al., 2015). Catalase treatment of highly metastatic cancer cell lines decreases migration and invasion (Liu et al., 2012). Due to their antioxidant activities and correlation with decreased aggressiveness in certain tumors, catalases have been envisaged as potentially important therapeutic agents (Glorieux et al., 2011; de Oliveira et al., 2016). For instance, a recent study shows that catalase activity correlates well with the ability of pancreatic cancers to resist chemotherapeutic H_2_O_2_ treatment with ascorbate (Doskey et al., 2016). In turn, altered levels of glutathione peroxidases are amply reported in cancer (Lu et al., 1997; Skrzydlewska et al., 2005), and the reduction potential of glutathione is associated with the proliferative and apoptotic state of a cell (Buettner et al., 2013). For instance, several glutathione peroxidases are upregulated in hepatocellular cancer (Carlson et al., 2012), and GPX4-deficient mice die shortly after birth (Carlson et al., 2016). GPXs are responsible not only for reducing H_2_O_2_ but also for reducing other ROS, such as lipid peroxides (Thomas et al., 1990; Esworthy et al., 1993). Peroxiredoxins have also been associated with tumorigenesis. PRDX1-4 and 6 display significantly altered levels in some prostate cancers (Basu et al., 2011; Whitaker et al., 2013). PRDXs may act as tumor suppressors (Egler et al., 2005), and their increased gene expression is associated with metastasis and aggressiveness (Park et al., 2006; Chang et al., 2007; Stresing et al., 2012). For instance, PRDX2 is highly expressed in lung metastases, and its knockdown decreases the formation of lung metastasis (Stresing et al., 2012). PRDX2 is also highly expressed in breast carcinoma, which correlates with the formation of lung metastases. PRDXs are often upregulated in cancers, and they contribute to cancer survival and resistance to oxidative stress (Lu et al., 2014) and radiotherapy (Wang et al., 2005). Interestingly, knockouts of the *PRDX* genes do not always result in a favorable outcome. For instance, *PRDX1* knockout mice show premature death, increased DNA oxidation and increased tumorigenesis and malignancy, and *PRDX3* knockout results in increased protein carbonylation in adipose tissues (see Cao et al., 2009; Perkins et al., 2014 and references therein). These observations show the crucial role of antioxidant enzymes in cancerogenesis and survivability.

Alterations in antioxidant enzymes and ROS levels occur throughout cancer progression. Overexpression of mitochondrial *SOD2* acts as a tumor suppressor in skin and breast cancers (Zhao et al., 2001; Robbins et al., 2012), suggesting that high SOD activity promotes tumor initiation. However, SOD levels also increase with tumor progression in these and other cancers (Ray et al., 2000; Chung-man Ho et al., 2001; Dhar et al., 2011; Miar et al., 2015), with similar changes occurring in other antioxidants, such as cytoplasmic SOD1 and catalase (Miar et al., 2015). Similarly, metastases show increased SOD2 protein levels when compared with matched tissues, less metastatic cell lines, or primary tumors (Liu et al., 2012; Miar et al., 2015). These changes are often accompanied by directly proportional changes in intracellular concentrations of H_2_O_2_, but they are not always followed by changes in other antioxidants. Changes in ratios of antioxidant proteins (e.g., SOD/catalase and SOD/GPX1) translate into differential intracellular concentrations of H_2_O_2_, and they vary with cancer stage and between metastatic and primary tumor cells (Miar et al., 2015). These observations show that while a comparison of the antioxidant expression levels between cancer and matched tissues is informative, such a comparison has to consider the developmental stage of the cancer.

Importantly, these antioxidant defenses are not found in all cell compartments, which should also differentially affect the responses of cancer cells to therapeutic targeting. For instance, mitochondria rely on superoxide dismutase 2, peroxiredoxin 3, thioredoxin 2, and thioredoxin reductase 2 for antioxidant defense and signaling transduction, but they do not rely on catalase (Rabilloud et al., 2001; Jones, 2006). Considering the crucial role that mitochondrial-generated ROS have in cancer initiation, progression, and apoptosis, the targeting of key antioxidant and redox signaling transduction systems in this compartment (e.g., PRDX3 Li and Yu, 2015) may have important consequences for global cell metabolism. However, it should be noted that targeting these antioxidants may lead to compensatory responses by the other antioxidants. For instance, knockdown of *PRDX3* promotes the upregulation of other peroxiredoxins, including those located in the cytoplasm (*PRDX1-2* and *PRDX6*; Li et al., 2008, 2013; Goncalves et al., 2012). This is particularly important because these proteins may catalyze similar reactions in H_2_O_2_ scavenging and disulfide exchange, albeit with different mechanisms and specificities (Perkins et al., 2014).

In addition, cancer cells cope with increased intracellular ROS by promoting the synthesis of compounds that enable their activity (e.g., glutathione for GPXs, Buettner et al., 2013 and TXNs for PRDXs, Arnér and Holmgren, 2006; Kaimul et al., 2007). GSSG/GSH ratios are higher in many cancers (Oltra et al., 2001; Skrzydlewska et al., 2005), in metastatic nodules and in circulating tumor cells (Piskounova et al., 2015), and the enzymes involved in GSH recycling or synthesis are often upregulated (Carlson et al., 2012; Harris et al., 2015; Lien et al., 2016) as response to oxidative stress. Mutations in PI(3)K/Akt, which are common in some cancer types, such as breast cancer, stabilize and activate NFE2L2, thereby promoting the upregulation of enzymes involved in synthesizing or reducing GSH (glutathione synthetase GSS, and glutathione reductase GSR; Lien et al., 2016). Upregulation of GSS and GSR is also associated with the increased resistance to oxidative stress observed in breast cancer. The inhibition of GSH biosynthesis sensitizes cancer cells to H_2_O_2_ and is potentiated by the utilization of other antioxidant inhibitors (Lien et al., 2016). High intracellular GSH concentrations also block drug-induced cytotoxicity in myeloma cells (Starheim et al., 2016). Cumulative evidence thus points toward GSH biosynthesis and homeostasis as a therapeutic target. However, in some cancers, inhibition of the GSH pathway alone does not prevent tumor progression. In addition to GSS, GSH is also synthesized by glutamate-cysteine ligase (GCL), an enzyme that consists of a heavy catalytic (GCLC) subunit and a light regulatory (modifier, GCLM) subunit. In mouse models of breast cancer, GCLM-deficiency or GLCM inhibition by Buthionine-[S,R]-sulfoximine (BSO) significantly prevented cancer initiation. However, this effect occurs only before tumor onset, and BSO treatment after onset does not alter tumor burden due to a compensatory role of TXN (Harris et al., 2015). GSH and TXN both serve as substrates for proteins with redox-important roles, including glutathione peroxidases, peroxiredoxins, glutathione and thioredoxin reductases, glutaredoxins, and sulfiredoxins (Björnstedt et al., 1994; Sun et al., 2001; Chang et al., 2004; Johansson et al., 2004; Peskin et al., 2016). Several cancers display increased expression of *TXN* and thioredoxin reductase 1 (*TXNRD1*) to compensate for GSH deficiency in *GCLM*^−/−^ cells (Mandal et al., 2010; Harris et al., 2015). The inverse is also observed, where *TXNRD1*-deficiency promotes the expression of *GSR* and *GCLC*, but not of *PRDX1, CAT*, or *SOD1*, in liver cancer cells (Carlson et al., 2012). Given that GSH and thioredoxins are relatively unreactive with ROS *per se* (Chae et al., 1994; Winterbourn and Metodiewa, 1999), the promotion of GSH or TXN biosynthesis is possibly promoting the peroxidase activities of GPX and PRDX.

*GLCM*-deficient cells also present increased expression of the cystine transporters and stabilizers *XCT* and *CD44* (Lu et al., 2015). This increased import is used toward promoting cysteine biosynthesis, which, in turn, is used toward TXN biosynthesis. Other observations show that chemotherapy treatment promotes *XCT* and *GCLM* expression with a concomitant increase in GSH biosynthesis in a HIF1-dependent mechanism related to therapeutic resistance (Lu et al., 2015). Targeting XCT, GCLM, and other pluripotency-involved transcription factors impaired malignant transformation. It is currently unclear whether other cysteine-based antioxidants, such as peroxiredoxins, also benefit from increased cystine import. Importantly, it was also observed that TXNs and thioredoxin reductases are upregulated and co-localize in several cancers, particularly in more aggressive cancers (Soini et al., 2001; Lincoln et al., 2003). These enzymes are associated with tumor initiation (Shen et al., 2016), and they correlated with worse prognosis (Cadenas et al., 2010). *TXNRD1* knockdown significantly slowed tumor progression and metastasis in lung carcinomas (Yoo et al., 2006), but it promoted cancer incidence in liver (Carlson et al., 2012).

Due to their chemical similarities and synergistic properties, recent approaches have simultaneously targeted both TXN and GSH metabolism, and they are significantly more effective at reducing tumor volumes than when they are applied individually (Harris et al., 2015). Multiple other studies have shown this efficacy in combination with common cancer drugs or radiotherapy for multiple cancers (e.g., Lu et al., 2007; Sobhakumari et al., 2012; Rodman et al., 2016; Roh et al., 2016; Tanaka et al., 2016). Together, the intracellular redox states expressed in terms of reducing equivalents and thiol compounds (Box 1) not only influence several signaling processes but also control the reactivities of ROS and their redox partners by regulating ROS homeostasis. It is currently unclear whether the involvement of some of these antioxidant defenses in cancer is related to their detoxification role, redox signaling properties or both. For instance, PRDX2 has increased levels in some cancer cells, which correlates with lower cytoplasmic H_2_O_2_ concentrations and cellular resistance to oxidative stress (Stresing et al., 2012). PRDX2 displays peroxidase activity and is also involved in redox signaling transduction (Neumann and Fang, 2007), such as the positive regulation of JNK-dependent DNA repair (Lee et al., 2011). Since these H_2_O_2_-scavenging and redox signaling properties stem from the kinetic properties of PRDX2/TXN/TXNRD1, GPX1/GSH/GSR, and catalase systems (Benfeitas et al., 2014; Tomalin et al., 2016), further studies are required to understand whether the culprit of PRDX2's tumorigenic association is its role as a peroxidase, chaperone, or redox signaling transducer. It is unclear whether the alterations in GSH/TXN biosynthesis and PRDX/GPX levels are more important toward controlling redox signaling, detoxification, or both. For instance, some observations indicate that sulfiredoxins and peroxiredoxins promote tumor growth and metastasis by modulating phosphokinase signaling cascades (Wei et al., 2011). Do these proteins directly interact with their targets? PTEN binds to PRDX1, but not to PRDX2, and this promotes Akt-mediated proliferation (Cao et al., 2009). The PRDX1-PTEN complex dissociates upon H_2_O_2_-mediated oxidation. A localized accumulation of H_2_O_2_, such as that occurring near cell membranes or near ROS sources, would thereby alter PTEN-mediated signaling transduction and proliferation by direct PRDX1 oxidation (Woo et al., 2010) or by relaying a redox signal from another more abundant, H_2_O_2_-reactive PRDX. PRDX2 is a good candidate as a H_2_O_2_ sensor due to its high reactivity with H_2_O_2_ (*k* ≈ 10^7^–10^8^ M^−1^ s^−1^), and it was recently observed to transmit oxidative equivalents to the transcription factor STAT3 (Sobotta et al., 2015), thereby controlling tumor proliferation and survival (Yu et al., 2014). While disulfide exchange between PRDX1 and 2 remains to be shown, the above observations indicate that the direct reactions of peroxiredoxins with transcription factors are important proliferative processes controlled by H_2_O_2_. It also remains to be seen whether the promotion of TXN biosynthesis (Harris et al., 2015), which is often linked to added ROS protection, is instead enabling secondary signaling transduction reactions, and whether multiple PRDX isoforms act synergistically in this process. Studying cancer ROS metabolism should therefore consider the toxicity of these oxidants and signaling disruption.

Overall, the observations above highlight important features that should be considered in cancer studies. First, targeting one antioxidant defense may elicit compensatory behaviors by other antioxidant defenses. Second, the close relationship between antioxidant proteins (e.g., GPX and PRDX) and their redox partners (e.g., GSH and TXN) requires that the choice of suitable therapeutic targets considers possible synergisms between them. Third, the high variability in responses, even for the same tumor, requires that cancer treatment is designed in a case- and stage-specific manner, rather than a cancer-type approach. Finally, all of these considerations need to be considered to identify antioxidant pathways that are differentially regulated by cancer, but not by normal cells. The targeting of antioxidant defenses as an approach for cancer treatment should therefore require tissue- and subject-specific phenotypic characterization.

## Energetic changes are coupled with maintenance of the antioxidant activity in cancer

Cancer cells display increased glycolytic activity and lower mitochondrial oxidative phosphorylation. Glucose uptake by breast, liver, colorectal, lung, and pancreatic cancers may reach 8–15 times the fluxes observed by surrounding normal tissues (see Boros et al., 1998 and references therein). This metabolic shift, characterized by increased ATP production from glycolytic pathways rather than respiratory pathways, even under aerobiosis, is one of the most well-known metabolic hallmarks of cancer cells, and it is generally referred to as the Warburg effect (Warburg, 1956). This process is crucial to maintaining the high energetic demand of fast proliferative cells. However, the energetic changes extend beyond the Warburg effect and are intimately related to the redox responses of cancer cells. This is the case of the metabolic changes that alter NADPH production. For instance, the MYC-controlled expression of pyruvate kinase type M2 (*PKM2*) is higher in cancer cells and promotes the diversion of carbohydrate metabolism from glycolytic pathways to other pathways (Vander Heiden et al., 2009), including the pentose phosphate pathway (PPP). Carbohydrates are thus diverted from ATP production to generate reducing equivalents and building blocks, such as phosphopentoses and ribonucleotides, supporting the fast proliferation of cancer cells (Boros et al., 1997; Raïs et al., 1999). Because ROS also regulate carbohydrate metabolism (Robbins et al., 2012; Hart et al., 2015; Miar et al., 2015) and some enzymes couple redox metabolism and ATP phosphorylation (Chang et al., 2004), the crosstalk between energetic and redox metabolism extends beyond enabling NADPH-driven peroxidase antioxidant activities.

Many of the glycolytic and PPP enzymes that are involved in NADPH production are elevated in cancer cells. Glucose-6-phosphate dehydrogenase (G6PD) and 6-phosphogluconate dehydrogenase (6PGD), which are both enzymes of the oxidative branch of PPP, catalyze the production of NADPH from hexoses entering the PPP from either glycolysis or the non-oxidative PPP. G6PD's activity is promoted by multiple oncogenic pathways upregulated in cancer (Stanton et al., 1991; Tian et al., 1994; Au et al., 2000; Wang et al., 2012; Zhang et al., 2014), and multiple studies have proposed that G6PD has pro-oncogenic activities (Wang et al., 2012; Patra and Hay, 2014; Zhang et al., 2014). *G6PD* overexpression leads to higher levels of intracellular NADPH, GSH, and nucleotide precursors, increased health span, and lower nucleotide oxidation (Nóbrega-pereira et al., 2016). These observations provide a clear link between PPP-mediated NADPH production and oxidative stress. In turn, G6PD deficiency severely limits cell resistance to oxidative stress (Pandolfi et al., 1995) and promotes oxidative damage to DNA (Jeng et al., 2013). These observations raise the hypothesis that a targeted inhibition of G6PD may be conducive to oxidative imbalance and ROS-mediated cell death. Considering that G6PD catalyzes the first and rate-limiting step of PPP and that it has a role in controlling the intracellular redox environment, this enzyme has been envisaged as one of the potentially most important therapeutic cancer redox targets (Wang et al., 2012; Patra and Hay, 2014; Zhang et al., 2014; Nóbrega-pereira et al., 2016), and it has been included in pre-clinical trials (Budihardjo et al., 1998; De Preter et al., 2015). *6PGD* is also upregulated in many cancers, including thyroid (Giusti et al., 2008), lung (Sukhatme and Chan, 2012), and cervical (Jonas et al., 1992) tumors. This enzyme is important for proliferation and tumor growth (Sukhatme and Chan, 2012; Shan et al., 2014), and its inhibition promotes senescence in lung cancer (Sukhatme and Chan, 2012). This phenotype results from altered glucose levels, but not altered NADPH levels (Sukhatme and Chan, 2012; Lin et al., 2015), suggesting that NADPH metabolism, and ultimately glucose metabolism, may adapt in such a way that compensates for the selective targeting of the PPP's enzymes. Interestingly, 6PGD suppression limits lipid biosynthesis and elevates intracellular ROS levels, and this effect translates into decreased tumor growth (Lin et al., 2015). However, conflicting observations regarding its importance in cancerogenesis (Sukhatme and Chan, 2012; Lin et al., 2015) also suggest that its role may vary depending on tissue and oncogenic background (Lin et al., 2015).

Other enzymes also promote downstream NADPH production. For instance, transketolase (TKT) and transaldolase (TALDO) are both enzymes of the non-oxidative PPP. While neither of them catalyze NADPH production, they are both important in directing the phosphorylated pentoses generated in the PPP back to glycolysis. Both enzymes are upregulated in cancer (Heinrich et al., 1976; Liu et al., 2010). TKT is required for cancer growth and controls resistance to oxidative stress by modulating NADPH levels. Its inhibition leads to higher intracellular ROS and decreased NADPH/NADP^+^ ratios (Xu et al., 2016), and it sensitizes cells to drug treatment. Importantly, *TKT* knockdown increases oxidative PPP fluxes, but it also leads to lower NADPH levels, which is a striking observation considering that NADPH is produced through the oxidative PPP; this point remains to be clarified. *TALDO*'s expression is linked to metastasis in hepatocellular carcinoma (Wang et al., 2011). TALDO deficiency also elicits hepatocellular carcinoma and promotes the formation of malignant tumors (Hanczko et al., 2009). These outcomes are associated with redox imbalances (lower NADPH and GSH levels) due to the insufficient recycling of PPP metabolites to support NADPH production, and they are reverted with dietary supplementation of antioxidants. This insufficient recycling exposes the liver to added oxidative stress and decreases lifespan. Other NADPH-producing enzymes are also upregulated in cancer cells, and some of the isoforms are exclusive to proliferating cells (Mazurek et al., 2005), suggesting that these enzymes may be selective therapeutic targets. Altered glycolytic and PPP metabolism has been proposed for potential therapeutic targeting in cancer (Wang et al., 2012; Patra and Hay, 2014; Zhang et al., 2014; Wen et al., 2015; Hay, 2016). Importantly, the crucial role of the PPP in cancer development seems to be associated with its redox homeostasis properties rather than its production of ribonucleotide precursors, as observed in hepatocellular carcinoma (Xu et al., 2016). Further studies are required to understand whether similar observations are present in other cancers.

While the oxidative PPP represents the main source of cytoplasmic NADPH in proliferating cells (Fan et al., 2014), other sources contribute significantly. Serine (Mehrmohamadi et al., 2014), folate (Tedeschi et al., 2013; Fan et al., 2014; Piskounova et al., 2015), and malate (Jiang et al., 2013) pathways also produce and regenerate NADPH and have crucial roles in maintaining the redox status and buffering oxidative stress in cancer cells. A system-wide comparison the contributions of these pathways to NADPH production and ROS metabolism in cancer is beginning to emerge (e.g., Tedeschi et al., 2013; Mehrmohamadi et al., 2014). In the context of antioxidant defense, most of these studies have focused on GSH-mediated ROS protection due to the close relationship between the PPP, serine/glycine metabolism, and *de novo* GSH biosynthesis. However, an analysis addressing the role of these pathways in supporting the activities of other important antioxidant defenses and redox signaling processes is currently missing. This becomes an important issue because defenses have different reducing equivalent requirements for activity, which also reflect their different antioxidant capacities and redox signaling roles. For instance, while antioxidant systems like PRDX/TXN/TXNRD and GPX/GSH/GSR stoichiometrically couple ROS detoxification to NADPH consumption, others, such as catalase, scavenge ROS while oxidizing virtually no NADPH. In normal cells, where energetic metabolism is limited, NADPH must be utilized toward lipid and cholesterol biosynthesis, and ROS decomposition by NADPH-consuming systems is thus an energetically expensive process. In cancer cells, where NADPH-producing fluxes are promoted, NADPH may be sufficiently abundant for cells to afford utilizing PRDX- or GPX-mediated detoxification. Importantly, the differences in the energetic requirements and kinetics of the PRDX/TXN/TXNRD, GPX/GSH/GSR, and catalase systems become particularly important if cancer treatments are targeting ROS metabolism by inhibiting NADPH production. ROS defenses may then be maintained by catalase or other energetically inexpensive processes, which is similar to what is observed in non-cancerous cells (Johnson et al., 2005; Benfeitas et al., 2014). Nevertheless, the lower cancer progression and increased ROS levels induced by the inhibition of NADPH-producing pathways indicate that this is a viable cancer therapy. A further understanding of the energetic requirements of antioxidant defense (e.g., PRDX/TXN vs. GSH/GPX pathways) may permit an efficient combination of anti-oxidant- and energetic-focused drug utilization for effective cancer treatment. Importantly, the heterogeneous gene expression and synergistic responses that may occur between alternative metabolic pathways at different cancer stages require an assessment of possible targets that considers specific oncogenic backgrounds.

## Systems biology and personalized medicine approaches are fundamental to revealing redox response in cancer

The observations above highlight the extensive crosstalk within and between ROS detoxification, redox signaling transduction, energy metabolism, and central metabolism. As such, the therapeutic targeting of cancer is more effectively strategized by addressing multi-pathway dysregulation (Pawson and Linding, 2008). Thus, while targeting the activity of specific enzymes may yield promising results *in vitro* and to a certain extent, *in vivo*, methods that encompass global metabolism are required to devise viable, cancer-specific treatment targets. Furthermore, these redox responses are highly heterogeneous, as has been observed by the different redox responses displayed by different cancer types, between individuals with the same cancer type, and between different cancer stages. Finding the best targets (Figure 4) and elucidating the mechanisms behind cancer phenotypes hence requires integrative analysis of a large number of biological networks, together with tissue- and patient-tailored data.

Systems biology aims at analyzing assorted biological data (e.g., genomics, proteomics, metabolomics, fluxomics), and it has consistently assisted in understanding the complex underlying mechanisms in health and disease (Mardinoglu and Nielsen, 2012; Agren et al., 2014; Benfeitas et al., 2014; Ghaffari et al., 2015b; Mardinoglu et al., 2017). Using systems biology approaches, others successfully clarified the role of small molecular decision circuits (e.g., Faratian et al., 2009; Gaglio et al., 2011; Tyson et al., 2011) and found commonalities across different cancers through pan-cancer analyses (e.g., Weinstein et al., 2013; Zack et al., 2013; Leiserson et al., 2014; Aran et al., 2015; Peng et al., 2015). However, few studies have addressed ROS metabolism using systems approaches (Zhang et al., 2010; Zhou et al., 2011; Zhan et al., 2012). For instance, modeling of the NFE2L2 pathway suggests that the high NFE2L2 expression that typically occurs in cancer cells promotes chemoresistance (Zhan et al., 2012) and suggests apparently opposite roles in antioxidant and ROS-mediated cancer signaling (Zhang et al., 2010). However, certain antioxidants, such as peroxiredoxins, display both scavenging and redox sensor and signaling transducer properties (Box 1). Mathematical modeling of H_2_O_2_ metabolism indicates that cancer-related transcription factors are unlikely to be activated by direct reaction with H_2_O_2_, and it points toward protein thiols as the likely signaling sensors and transducers. Others have observed that the glutathione and NADPH synthesis pathways are simultaneously up- or down-regulated in breast, ovary, colon and lung cancers, establishing important interactions with *de novo* nucleotide synthesis (Mehrmohamadi et al., 2014), which suggests that cancers utilize redox homeostasis and biosynthesis pathways in parallel. ROS and redox responses of cancer cells have yet to be more extensively examined.

With the advent of big data, recent approaches aimed at understanding cell metabolism now incorporate large reaction networks derived from omics technologies (genomics, transcriptomics, proteomics, and others). By encompassing whole-cell reaction networks, GEMs have helped identify important redox alterations in metabolic diseases and physiological processes. For instance, disturbed H_2_O_2_ metabolism is observed (Mardinoglu et al., 2017) in non-alcoholic fatty liver disease, specifically due to deficient GSH biosynthesis (by GCLC/GCLM, GSR) and NADPH production (NNT). A lower abundance of plasma glycine, a substrate for *de novo* GSH biosynthesis, is also found in subjects with high hepatic steatosis. Observations in mice (Mardinoglu et al., 2015b) indicate that commensal gut microbes decrease glycine availability in the gastro-intestinal tract of the host, which results in decreased *de novo* GSH synthesis and promotes *NNT* and *GSR* expression, possibly to compensate for the decreased GSH pool. In the context of cancer, publicly deposited genomic data permit the stratification of cancer patients based on network-specific mutations (Hofree et al., 2013), and these data have been used to find biomarkers and potential cancer therapeutic targets (Jerby and Ruppin, 2012; Agren et al., 2014). Others have combined experimental and interactome data with stochastic modeling to find that ROS and DNA damage are necessary and sufficient for senescent growth arrest (Passos et al., 2010). Chronic, non-toxic ROS supplementation reverses drug resistance in carcinoma cells (Maiti, 2010), which, through pathway analysis, identified several genes (e.g., *TP53*, Rac/Cdc42 guanine nucleotide exchange factor 6 *ARHGEF6*, and a DNA-activated protein kinase *PRKDC*) that mediate ROS-related apoptosis. Reconstruction of a generic human whole-cell GEMs encompassing >9,000 reactions and >3,000 metabolites and genes (Mardinoglu et al., 2014a) enabled context-specific integration and interpretation of cancer omics data. Comparisons between hepatocellular carcinoma and healthy liver samples using these models indicate that tumors display an increased abundance of NADPH-producing enzymes (e.g., ME1, G6PD, TALDO1, and TKT) and that H_2_O_2_ may be used as reporter metabolite in some of the patients (Bjornson et al., 2015). Personalized medicine approaches would greatly benefit from clarifying redox cancer responses. GEMs were previously used to identify novel anticancer drugs by reconstructing patient- and tissue-specific GEMs (Agren et al., 2014) and to identify anti-growth factors in human cancer cell lines (Ghaffari et al., 2015a). Cell-specific GEMs have also been used to identify malonyl-CoA decarboxylase, an important enzyme in fatty acid metabolism, as a selective and effective cancer therapeutic target (Yizhak et al., 2014a). Its inhibition drains reducing equivalents, decreases GSH/GSSG ratios, and promotes oxidative stress, which may help in chemotherapeutic approaches. Patient-specific breast and lung cancer GEMs were also able to predict patient survivability, and they predicted that knockout of *GSR* significantly affects cancer growth.

**Figure 6:**
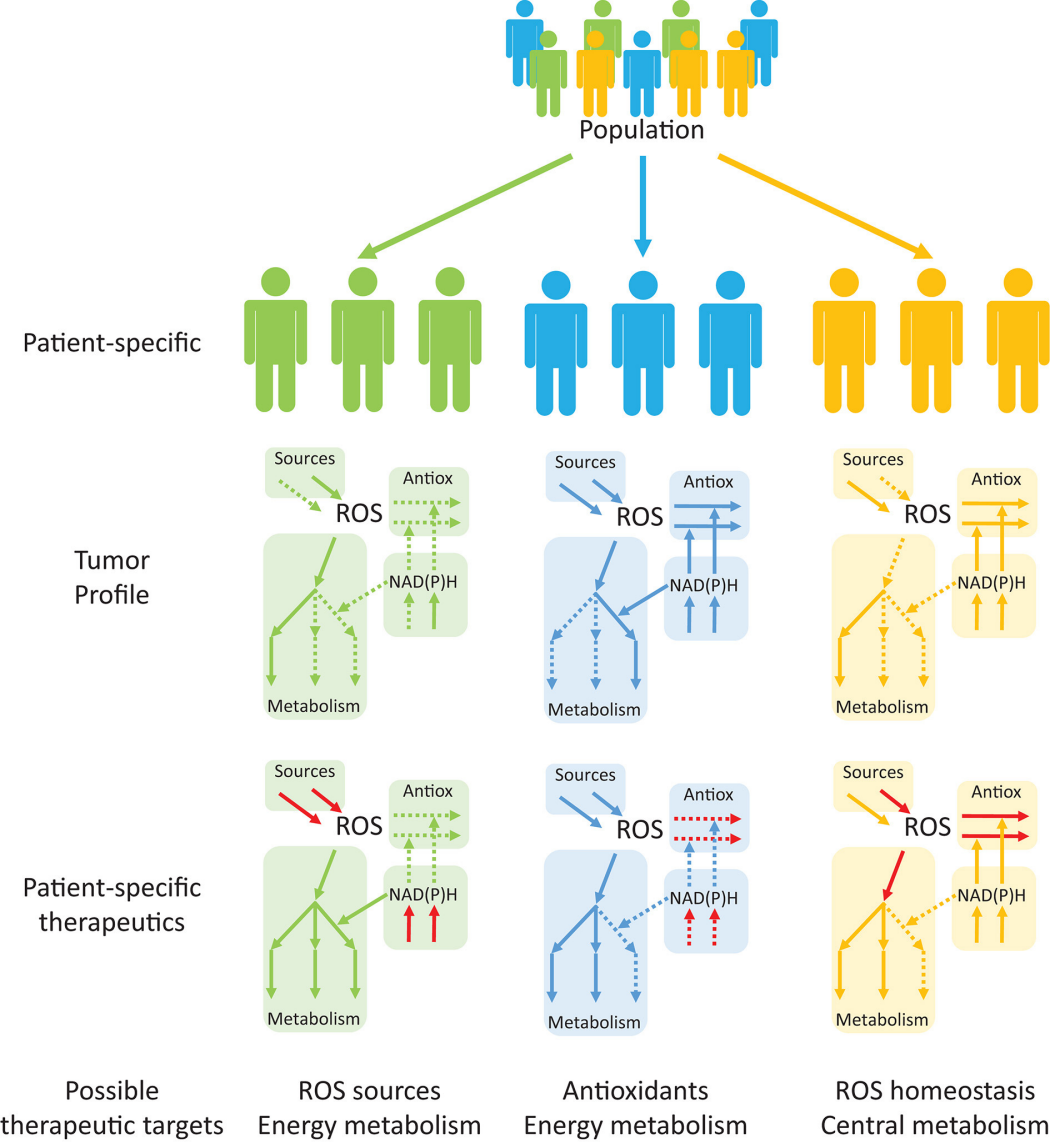
Personalized systems medicine approaches are emerging as useful tools in devising patient-specific, rather than population-based, therapeutic targets in cancer. Tumor profiling of patients may help in identifying up- and down-regulated pathways (continuous and dashed arrows, respectively) that are suitable for therapeutic targeting. Drug targeting of specific processes (red arrows), either to promote or inhibit the processes, will permit alterations in the consequences of redox processes in cancer and other diseases.

Interestingly, despite an enzyme- or process-specific focus in redox cancer metabolism in recent years, system-wide studies of ROS and redox metabolism and their interactions with central metabolism in multiple cancers are lacking. Antioxidant profiling of tumor cells and their surrounding cells may be used in conjunction with patient-specific GEMs to identify the best therapeutic targets in this disease (Figure 6). Studies using reaction network information with tissue- and patient-specific models will be useful in (1) Redox profiling of patient-specific cancer tissues; (2) Understanding mechanistic properties of redox responses; (3) Devising effective, selective and patient-specific therapeutic strategies to regulate redox responses; (4) Establishing redox-based therapies to synergize with existent drugs (Kasiappan and Safe, 2016) and identifying and averting drug-resistance mechanisms; and (5) Shortening the gap between pre-clinical and clinical trials, potentially overcoming issues faced by previous trials, such as those that assessed the impact of dietary antioxidants on cancerogenesis (Omenn et al., 1996; Goodman et al., 2011; Klein et al., 2011; Sayin et al., 2014). The adopted strategies (Figure 6) will benefit from patient-specific tumor profiling to identify single or multiple targetable processes within the same pathway or to identify processes that serve as metabolic central hubs. The combination of these approaches with drugs that target other metabolic processes may promote desirable synergisms. Redox systems medicine is thus an interesting emerging field with potentially important implications for disease treatment.

## Conclusion

Redox, energetic and central metabolism are closely intertwined, and the view that ROS are simple secondary products of cell metabolism is long gone. Together with their redox partners, ROS and antioxidant defenses are now regarded as crucial processes in tumorigenic initiation, progression and aggressiveness. However, the redox cancer responses are highly heterogeneous, manifesting not only between different cancer types but also between patients who suffer from the same cancer, and they are altered throughout cancer progression. The influence of ROS on different biochemical levels makes it necessary to seek an integrative analysis of these systems at the genomic, proteomic, and metabolomic levels. Approaches that are able to encompass these levels and integrate the crosstalk between antioxidant, redox, energetic, and central metabolism are able to capture and understand these complex responses. Systems biology approaches may be used to analyze omics data and understand the roles of each redox system in cancer. These approaches may be tissue- and patient-tailored, which enables the identification of the best therapeutic targets, while taking in account patient-specific oncogenic backgrounds. This is the aim of personalized systems medicine (Mardinoglu and Nielsen, 2015; Schork, 2015), an emerging field that presents a high potential to overcome some of the problems in therapeutic treatments, including the low (< 25%) drug efficacies caused by population—rather than patient-wise data (Schork, 2015). Together with existing drugs, novel or existing redox-targeting drugs may be identified to produce synergistic responses for the treatment or prevention of cancer. Personalized medicine may thus enable an understanding of the role of redox systems in cancer and other diseases and may assist in drug discovery.

## Author contributions

RB has written the manuscript. All authors actively contributed in writing and editing of the manuscript.

### Conflict of interest statement

The authors declare that the research was conducted in the absence of any commercial or financial relationships that could be construed as a potential conflict of interest.
